# Omega-3 Fatty acids as Monotherapy in Treating Depression in Pregnant Women: a Meta- Analysis of Randomized Controlled Trials

**Published:** 2017

**Authors:** Liu Wei-Hong, Zhang Cheng-Gui, Gao Peng-Fei, Liu Heng, Yang Jian-Fang

**Affiliations:** a *The Key Laboratory of Medical Insects and Spiders Resources for Development & Utilization at Yunnan Province, Dali University, Dali 671000, Yunnan Province, China.*; b *The Libraries of Dali University, Dali 671000, Yunnan Province, China. *; c *National-local Joint Engineering Research Center of Entomoceutics, Dali 671000, Yunnan Province, China. *; d *School of Foreign Languages, Dali University, Dali 671000, Yunnan Province, China.*

**Keywords:** Major depressive disorder, MDD, Omega-3 fatty acids, Meta-analysis, Women

## Abstract

Previous studies have reported inconsistent findings regarding the efficacy of omega-3 fatty acids on pregnant women with major depressive disorder (MDD). This meta-analysis was conducted to systematically evaluate the clinical applicability of omega-3 fatty acids in treating depression in pregnant women.

Randomized controlled trials (RCTs) that compared omega-3 fatty acids to placebo for short-course treatment of depression in pregnant women were systematically reviewed between March 1999 and April 2015. The search terms used were ‘depression’, ‘omega-3 fatty acids’, ‘fish oil’, ‘eicosapentaenoic acid’ and ‘docosahexaenoic acid’. Standardized difference in means of depression scale was used as the main outcome. Random effect model was used. The effects of baseline depression scores were studying by meta-regression analysis.

patients received omega-3 fatty acids. The pooled standardized difference in means was 0.75 with 95% CI= (0.47, 1.04). The baseline depression scores had no effect on the efficacy. None of the recruited patients was withdrawn.

## Introduction

Major depressive disorder (MDD) is one of the most prevalent debilitating mental disorders, which puts heavy disease burden on individual, family, and society (1). MDD affects about 10% of the population (2), and women have higher risk than man. Due to the body changes caused by pregnancy, many women suffer from MDD during this time (3). Previous studies showed that MDD could affect 10~ 20% of the perinatal women (4, 5). If untreated, MDD can increase the risk of negative pregnancy outcomes (6). But, up to now, although many researchers have used metabonomics to successfully identify some potential biomarkers for diagnosing unipolar depression (7, 8) and bipolar disorder (9-11). there is still no objective laboratory test method to diagnose MDD. Moreover, as far as we know, none of the currently available antidepressant could treat MDD with 100% response rates. Repetitive transcranial magnetic stimulation could obtain about 40%~48% response rates (12, 13), and the response rates of electroconvulsive therapy varied from below 20 to 70% or higher (14, 15). Generally speaking, about 30% of the patients receiving treatment fail to respond to the first-line treatments (16). Additionally, some researchers have concerns that selective serotonin reuptake inhibitor antidepressants could increase the risk of perinatal complications (17) and have side-effects on the fetus (18). Therefore, there is an urgent need to find a novel and safely treatment for depression in pregnant women. 

Omega-3 fatty acids are nutritional compounds that could not be synthesized by the human body (19). Some clinical studies showed that the deficit of omega-3 fatty acids might increase the incidence of MDD (20-22). Researchers also found the decreased omega-3 fatty acids levels in patients with MDD (23). Therefore, researchers assumed that omega-3 fatty acids might have antidepressant efficacy, and completed many meaningful works. Michael *et al*. found a small, non-significant benefit of omega-3 fatty acids for MDD (24). Freeman *et al.* found the significant benefit of omega-3 fatty acids over placebo in treating affective disorders (25). Linda *et al*. suggested that whether omega-3 fatty acids administration was effective in the treatment of perinatal depression or not needed future studies to answer (26). Meanwhile, pregnancy period provides an excellent opportunity to examine the relationship between omega-3 fatty acids and MDD. Therefore, we did this meta-analysis to assess the efficacy of omega-3 fatty acids as monotherapy in treating depression in pregnant women. The results of this work will help clinicians to make an optimal treatment method for pregnant women with MDD. 

## Experimental

*Literature reviewing*

Electronic searches were conducted in several international databases, such as PubMed, CCTR, Web of Science, and Embase, and two Chinese databases (CBM-disc, CNKI) between March 1999 and April 2015. The search terms used were ‘depress*’ combined with ‘omega-3 fatty acids’, ‘fish oil’, ‘eicosapentaenoic acid’ and ‘docosahexaenoic acid’. No language restriction was imposed. Studies met the following inclusion criteria were used for the subsequent analysis: (i) randomized controlled trials (RCTs) (ii) omega-3 fatty acids versus placebo (active or not); (iii) depression in pregnant women over 18 y of age; (iv) provided informed consent and (v) outcome assessed by depression scales, such as Hamilton Depression Rating Scale (HDRS), Clinical Global Impression (CGI), Montgomery–Åsberg Depression Rating Scale (MADRS), Geriatric Depression Scale (GDS) or Beck Depression Inventory (BDI). Meanwhile, studies met any following criteria were excluded: (i) duplicate studies; (ii) nonrandom allocation; (iii) depression in men and (iv) case reports and reviews. 

**Figure 1:**
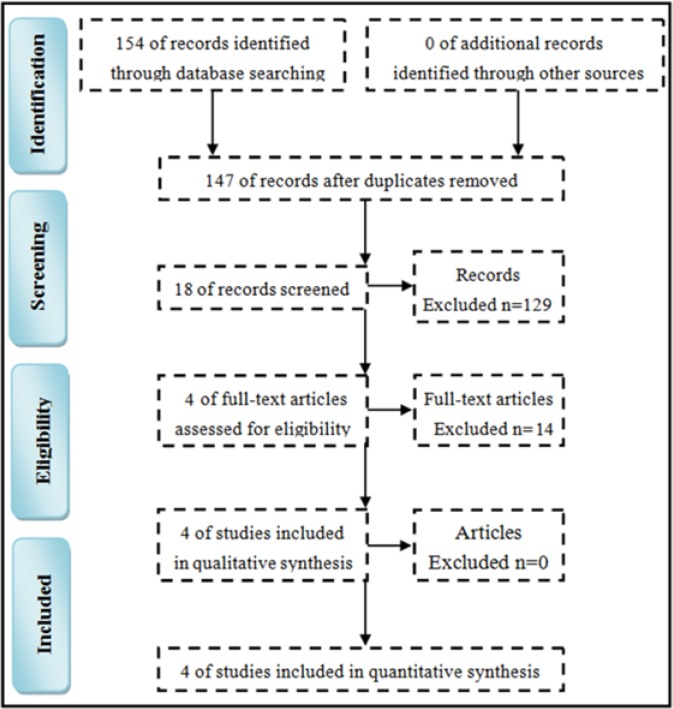
Literature search

**Figure 2:**
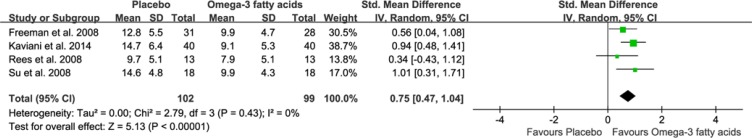
Meta-analysis of omega-3 fatty acid versus placebo

**Figure 3:**
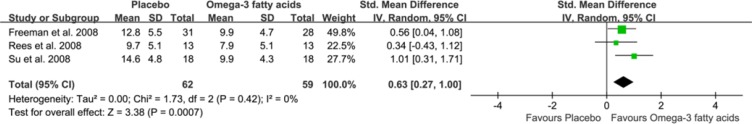
Sensitivity analysis of omega-3 fatty acid versus placebo

**Table 1:** Clinical characteristics of the patients

**RCT**	**Subjects, n**	**Age, ** **(S.D** **.** **)** **(I/C)**	**Depression scale**	**Patient status**	**E** **thnic group**
Kaviani *et al*. 2014	80 (40/40) (I/C)	26.3(4.2)/25.2(4.2)	BDI-21	pregnancy	Iran
Freeman *et al*. 2008	59 (31/28) (I/C)	31.0(5.8)/29.7(6.2)	HDRS,CGI, EPDS	pregnancy	United States
Rees *et al.* 2008	26 (13/13) (I/C)	31.2(4.4)/34.5(3.8)	HDRS,MADRS, EPDS	pregnancy	Australia
Su *et al*. 2008	36 (18/18) (I/C)	30.9(3.9)/31.3(5.7)	HDRS, EPDS,BDI-21	pregnancy	China

RCT: randomized controlled trial; I/C: intervention/control; BDI: Beck Depression Inventory; HDRS: Hamilton Depression Rating Scale; CGI: Clinical Global Impression; EPDS: Edinburgh Postnatal Depression Scale; MADRS: Montgomery- Åsberg Depression Rating Scale; MDD: major depression disorder.

**Table 2.** Parameters of the treatment method.

**RCT**	**Intervention**	**S** **trategy**	**Control**	**Daily dose**	**Time**
Kaviani *et al*. 2014	omega-3 fatty acids	Monotherapy	olive oil	1g	6 weeks
Freeman *et al.* 2008	omega-3 fatty acids	Monotherapy	corn oil+1% fish oil	1.1 g EPA+0.8 g DHA	8 weeks
Rees *et al.* 2008	omega-3 fatty acids	Monotherapy	sunola oil	0.42 g EPA. 1.64 g DHA	6 weeks
Su *et al*. 2008	omega-3 fatty acids	Monotherapy	olive oil	2.2 g EPA 1.2 g DHA	8 weeks

RCT: randomized controlled trial; DHA, docosahexaenoic acid; EPA, eicosapentaenoic acid.

**Table 3:** Bias risk of the included randomized controlled trials

**Study**	**Randomization**	**Blinding**	**Allocation**	**Baseline**	**Incomplete data**
Kaviani *et al*. 2014	None	None	None	None	None
Freeman *et al*. 2008	None	None	None	None	None
Rees *et al*. 2008	None	None	None	None	None
Su *et al*. 2008	None	None	None	None	None

*Bias Risk Assessment*

The quality of the suitable RCTs was independently assessed by two authors according to the Cochrane Collaboration criteria. The items, such as the randomization, allocation concealment, blind assessment and incomplete data were used to assess the bias risk. 

*Data Extraction*

After literature reviewing, two authors independently checked the obtained studies according to the inclusion/exclusion criteria. The two authors extracted the data from the suitable studies. Any disagreement was solved by consulting with a third reviewer. We extracted the following data from the suitable studies: (i) patients’ characteristics (i.e., number of patient, mean age, patient status, country, and depression scale); (ii) parameters of the treatment method (i.e., treatment time, strategy, placebo and daily dose); and (iii) bias risk of the included studies. When the RCTs reported results from kinds of depression scales, HDRS was preferentially selected. Good faith efforts were applied to obtain the data that could not be directly extracted from the RCTs.

*Statistical Analysis*

Statistical analysis was conducted using Revman 5.1 and SPSS 19.0. The means and standard deviation (SDs) of depression scale before and after treatment in two groups were extracted. We used these data to do pooled analysis (27), and calculated the standardized mean effect by using Hedges adjusted *g *to correct the small sample bias (28). Mantel-Haenszel random-effects model was used.

Heterogeneity was assessed using I^2^ and Q statistic test (29). The Egger›s test and funnel plots were used to assess the publication bias. Meta-regression analysis was used to assess the effects of the baseline depression scores. The protocol of this work followed the recommendations for conducting a meta-analysis (30). 

## Results

*Workflow of literature search*

The search was according to the Preferred Reporting Items for Systematic Reviews and Meta-Analyses (PRISMA) guidelines (31). Totally, the initial internet search yielded 154 potentially relevant studies. After removing the 7 duplicate studies, 147 studies remained. Among these, 89 studies were excluded by reviewing the titles, 40 studies were excluded by reviewing the abstract and 14 studies were excluded by two authors independently reviewing the full texts. Finally, four RCTs met all the aforementioned criteria and were used for this meta-analysis (Figure 1).

*Description of included studies*

Totally, these four studies recruited 201 pregnant women with MDD, composed of 102 patients receiving placebo and 99 patients receiving omega-3 fatty acids (32-35). The average age of these patients was about 30 y old. These four studies were from different countries. Two studies used olive oil as placebo; one study used corn oil plus 1% fish oil as placebo; another study used sunola oil as placebo. All the included studies used omega-3 fatty acids as monotherapy to treat patients with six or 8 weeks. The detailed information was described in Table 1 and Table 2.

*Bias risk of included studies*

All the included studies conducted adequate randomization and allocation concealment. The raters and patients were blinded to the treatment methods. The baseline characteristics of patients in two groups were similar. Incomplete data, if existed, was reported by all studies. Generally speaking, the double-blind was difficult to carry out in clinical study. But all the four studies successfully blinded the raters and patients. Thus, these four studies in this meta-analysis were consistently high-quality and displayed no bias risk (Table 3).

*Meta-analysis*

Four studies were used to perform meta-analysis. The pooled standardized difference in means was 0.75 (95% CI=0.47, 1.04) for the random-effects model (Figure 2), which indicated a beneficial effect of the omega-3 fatty acids for pregnant women with MDD compared with placebo. Moreover, heterogeneity in effect size was very low *(P*=0.43, I^2^=0%). Meanwhile, meta-regression analysis was conducted to assess the effect of baseline depression scores on the efficacy of omega-3 fatty acids. The results showed the negligible relation between the baseline depression scores and the efficacy (regression coefficient = 0.021, 95% CI=-0.009, 0.033; *P*=0.37). The inverted funnel plots of these RCTs showed no significant asymmetry. As the total number of studies was too limited to show clear asymmetry, we also performed the Egger’s test. The results (t=-1.54, *P*=0.27) showed the outcome was not influenced by publication bias.

Three studies mainly used HDRS to assess the depressive symptoms, and one study used BDI-21 to assess the depressive symptoms. Therefore, we did sensitivity analysis by excluding this study. This exclusion did not significantly affect the initial effect-size estimates for the whole sample. The pooled standardized difference in means was 0.63 (95% CI=0.27, 1.00) for the random-effects model (Figure 3). Still, no significant heterogeneity was existed (*P*=0.42, I^2^=0%).

## Discussion

As far as we know, this is the first meta-analysis to study the efficacy of omega-3 fatty acids as monotherapy in the acute treatment of MDD in pregnant women. The results showed that the omega-3 fatty acids as monotherapy produced better efficacy than placebo with standardized difference in means of 0.75 (95% CI=0.47, 1.04). No significantly heterogeneity in effect size was existed. Concerning the acceptability, none of the included patients withdrawn and experienced serious side-effects. Meanwhile, the sensitivity analysis yielded the similar results. Based on these results, omega-3 fatty acids as monotherapy might have a beneficial effect in depression during pregnancy. Considering the safety issue and psychotherapeutic effect for newborns and mothers, the clinical applicability of omega-3 fatty acids showed greater promise and should be further explored. Limited by the small number of the included studies and patients, this conclusion should be interpreted with caution and needed future studies to confirm.

Some researchers proposed omega-3 fatty acids as a novel and potential treatment method for MDD (36). However, previous studies have demonstrated inconsistent findings regarding the efficacy of omega-3 fatty acids vs. placebo in treating depression in pregnant women. Kaviani *et al*. reported that using omega-3 fatty acids was a suitable method for treating mild depression during pregnancy (35). Su *et al*. found that omega-3 fatty acids might have antidepressant efficacy for pregnant women with MDD (33). But other studies reported that there was no benefit for omega-3 fatty acids over placebo in treating depression in pregnant women (32, 34). These discrepant conclusions might be caused by the relatively low statistical power among some of the individual studies (37). Therefore, we applied meta-analytical approaches to examine the efficacy of omega-3 fatty acids in pregnant women with MDD. By integrating the findings from multiple studies, the results in this work obtained by this approach should be more accurate and robust (38). 

Several limitations should be mentioned here: i) the relatively small number of included studies and depression in pregnant women; ii) the recruited studies had the different population, placebo and dose, which were also the general problems for meta-studies to solve; iii) publication bias, which was difficult to detect and somewhat was controversial when the number of included studies was small (39). However, this pooled analysis of four high-quality double-blinded RCTs found that omega-3 fatty acids was a potential treatment method for pregnant women with MDD.

## Conclusion

The abovementioned results showed that omega-3 fatty acids could produce a beneficial effect on depression in pregnant women compared with placebo. The clinical applicability of omega-3 fatty acids showed greater promise and should be further explored. 
